# Trends in ethnic inequality in child welfare interventions in Wales, 2010 – 2021

**DOI:** 10.23889/ijpds.v8i2.2336

**Published:** 2023-09-14

**Authors:** Yongchao Jing, Sin Yi Cheung, Lucy Griffiths, Jonathan Scourfield

**Affiliations:** 1 Cardiff University, Cardiff, United Kingdom; 2 Swansea University, Swansea, United Kingdom

## Objectives

Children’s chances of receiving welfare interventions are found to vary by ethnicity in England, but the ethnic pattern in child welfare interventions in Wales over time is unknown. We aim to estimate the scale and trend of ethnic inequalities in intervention rates in Wales over a 12-year period, using population-based linked administrative records.

## Methods

We analyse cross-sectional administrative children’s social care data: the Children in Need (CIN) dataset from 2010 to 2016 and the Children Receiving Care and Support (CRCS) dataset from 2017 to 2021. These data are linked to both the ethnic population data to obtain the ethnic variation in child welfare intervention rates and, using the WIMD, to identify the relative deprivation level in the neighbourhoods from which children entered care. For observations in CIN/CRCS whose ethnicity are missing, we link Census 2011 to obtain the children’s ethnicity to achieve a fuller coverage. For the first time, our analysis also links children’s social care data with the religion variable in the Census data, allowing us to estimate the extent of religious inequalities in child welfare intervention.

## Results

Based on research findings in England on ethnic variation in child welfare intervention, we hypothesise higher intervention rates among Black children and a lower intervention rates among Asian children in Wales, compared to White children, controlling for deprivation status. By extension, we also hypothesise lower intervention rates among Muslim children compared to Christian children.

## Conclusion

No research to date has quantitatively documented the pattern and trend of ethnic inequalities in child welfare intervention in Wales using linked administrative data on CIN/CRCS. Our study is also the first to examine religious inequality in UK child welfare by linking social care data to the Census. Our findings will have significant implications on policy and practice in social work and particularly in children’s social services. We conclude by discussing what future research questions may emerge from the new insight we shed.

